# Planar and Helical Dinaphthophenazines

**DOI:** 10.1021/acs.joc.2c00129

**Published:** 2022-05-26

**Authors:** Fengkun Chen, Manuel Melle-Franco, Aurelio Mateo-Alonso

**Affiliations:** † POLYMAT, University of the Basque Country UPV/EHU, Avenida de Tolosa 72, 20018 Donostia-San Sebastian, Spain; ‡ Department of Chemistry, CICECO—Aveiro Institute of Materials, University of Aveiro, 3810-193 Aveiro, Portugal; § Ikerbasque, Basque Foundation for Science, 48009 Bilbao, Spain

## Abstract

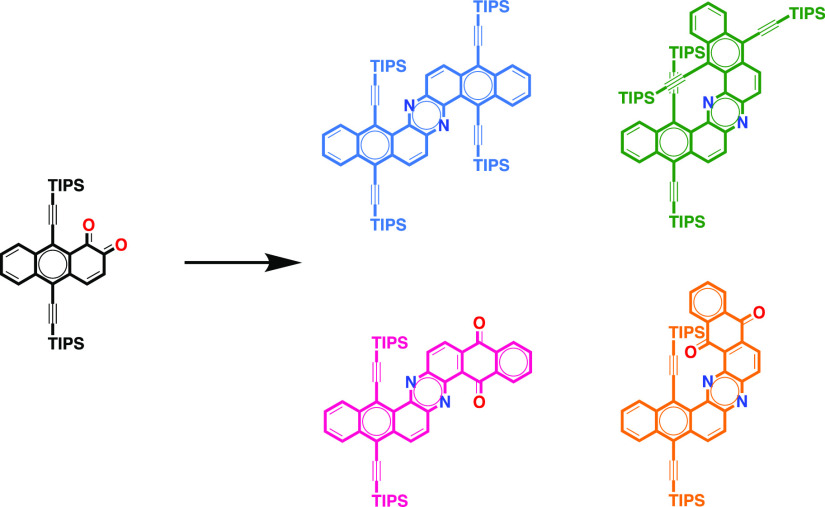

In this study, we report the synthesis of a series of planar and helical dinaphthophenazines by cyclocondensation reactions between the newly developed 9,10-bis((triisopropylsilyl)ethynyl)anthracene-1,2-dione and different diamines. Their optoelectronic and electrochemical properties are studied by ultraviolet–visible (UV–vis) spectroscopy, fluorescence spectroscopy, cyclic voltammetry, and density functional theory calculations.

## Introduction

Nonplanar polycyclic aromatic hydrocarbons (PAHs) and nanographenes possess particular optoelectronic properties that are derived from their nonplanar π-conjugation and unique intermolecular π-contacts.^1^

**Figure 1:**
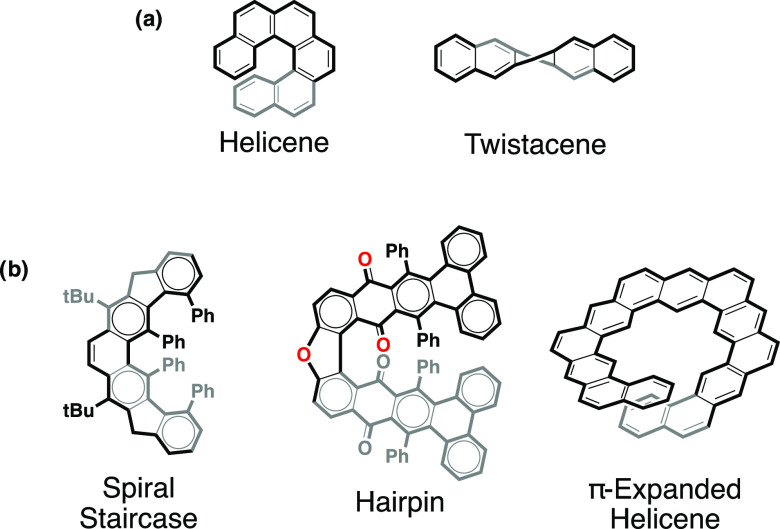
(a) General structure of helicenes and twistacenes. (b) Examples of previously reported hybrid helical aromatics, including a molecular “spiral staircase,”^5^ a molecular “hairpin”,^6b^ and a π-expanded helicene.^7c^

Helicenes^1e−1h^ and twistacenes^1d,1g,1h^ (Figure 1a) are some of the most representative nonplanar PAHs and have attracted considerable attention as optoelectronic materials for polarized light emitters and detectors,^2^ nonlinear optics^3^ and spintronics,^4^ etc. Helicenes and twistacenes are helical systems that differ in the direction of the helix propagation axis that is imposed by the arrangement of the rings in the aromatic core (Figure 1a). Helicenes consist of angularly ortho-annulated rings in a helical arrangement along the axis perpendicular to the rings, as a result of the steric interactions between terminal aromatic rings, while twistacenes consist of linear ortho-annulated rings and exhibit a helical structure along the axis parallel to the rings, as a result of the steric interactions between sterically demanding peripheral substituents.

**Chart 1:**
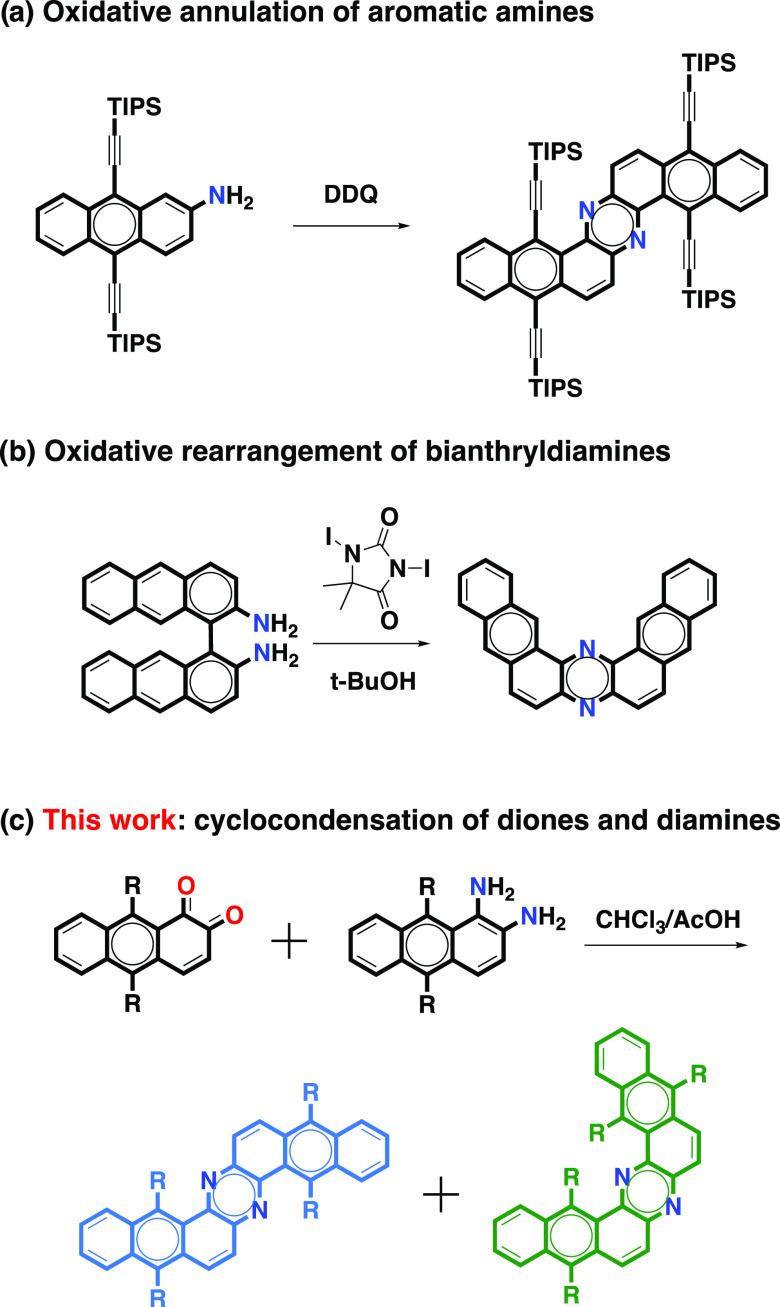
Different Approaches to Dinaphthophenazines

There is another class of helical aromatics that combines both angularly and linearly annulated rings. Examples of these include the molecular spiral staircase,^5^ molecular hairpins,^6^ and π-expanded helicenes^7^ (Figure 1b). In these terms, dinaphthophenazines are a family of compounds that combine linear and angular annulations and that have received little attention. The different arrangements of their fused rings can give rise to Z-shaped (dinaphtho[*a*,*h*]phenazine) or U-shaped (dinaphtho[*a*,*j*]phenazine) isomers. However, there is a limited number of methods to obtain these structures. The Z-shaped dinaphthophenazine has been obtained by the oxidative annulation of aminoanthracenes (Chart 1a).^8^ Meanwhile, the U-shaped dinaphtho[*a*,*j*]phenazine has been obtained by the oxidative rearrangement of bianthryldiamines (Chart 1b).^9^ In both cases, the routes yielded planar dinaphthophenazines. However, the U-shaped isomers, if properly functionalized, can give rise to π-expanded helicenoids.^10^

Cyclocondensation reactions between *o*-quinone and *o*-diamine precursors have been widely used for the construction of nitrogen-doped PAHs, nanographenes, and two-dimensional (2D) polymers.^11^ However, despite these advances and the possibilities offered by cyclocondensation reactions to prepare nonplanar systems, this approach has not been explored in the synthesis of dinaphthophenazines. Herein, we describe a synthetic route for the synthesis of different dinaphthophenazines by means of cyclocondensation reactions (Chart 1c). This route provides a mixture of the Z- and U-shaped structural isomers that can be isolated by chromatography. The Z-shaped dinaphthophenazines are almost planar because there is no steric interaction between the bulky triisopropylsilyl (TIPS) substituents. Whereas, the U-shaped dinaphthophenazines adopt a π-expanded helicene structure as a result of the steric interactions between TIPS groups that point to the center of the helix.

## Results and Discussion

**Scheme 1:**
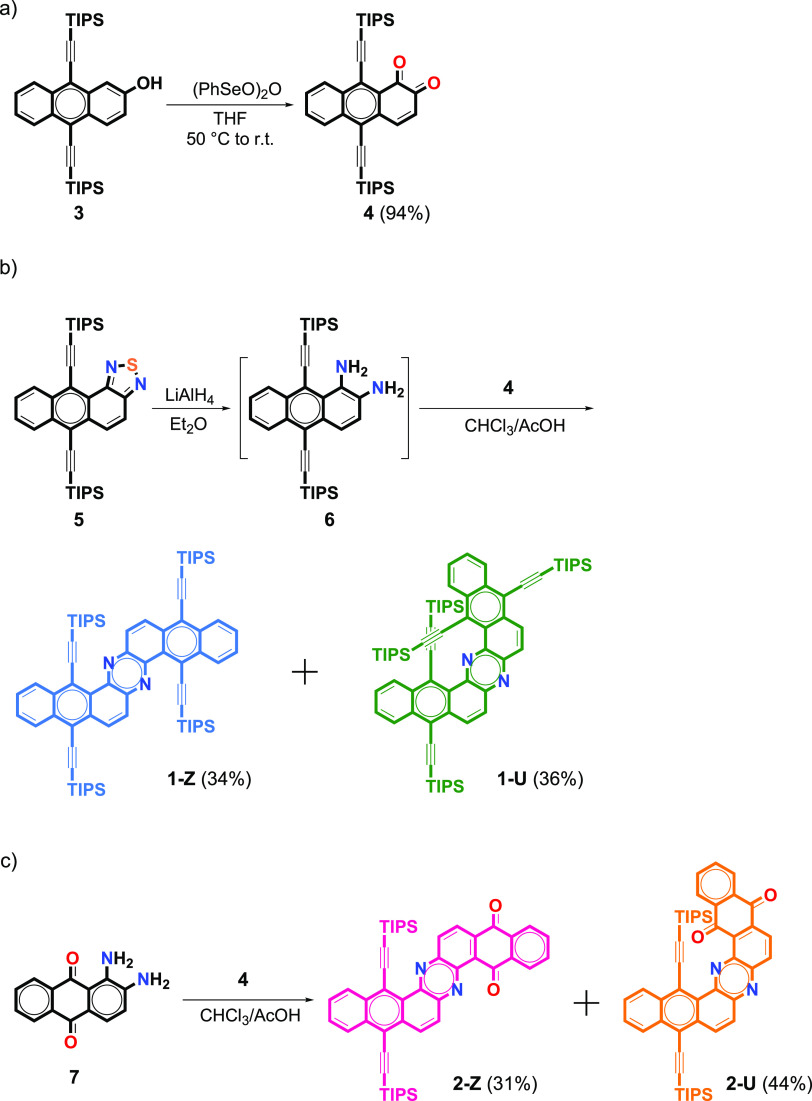
Synthetic Routes

The synthesis of the target dinaphthophenazines **1** and **2** requires the synthesis of appropriate *o*-dione and *o*-diamine building blocks (Scheme 1). Anthracene-1,2-dione **4** was synthesized from 2-hydroxy-9,10-bis(triisopropylsilylethynyl)anthracene **3**, as shown in Scheme 1a. Treatment of **3** with phenylseleninic anhydride as an oxidant provided **4** in a 94% yield. Diamine **6** was generated by reduction of **5** with LiAlH_4_ in diethyl ether at 0 °C. Due to the limited stability, amine **6** was not purified and it was used directly in the next step. The cyclocondensation of amine **6** with dione **4** in a 1:1 mixture of AcOH/CHCl_3_ in refluxing conditions afforded **1-Z** and **1-U** with yields of 34% and 36%, respectively, both as yellowish solids after chromatographic purification (Scheme 1b).

To expand the library of dinaphthophenzines and to test the versatility of the precursors, dione **4** was condensed with 1,2-diaminoanthracene-9,10-dione **7** under the same conditions mentioned above (Scheme 1c). The two structural isomers, namely, **2-Z** (31%) and **2-U** (44%), were obtained as red solids after purification by column chromatography.

The structures of **1-Z**, **1-U**, **2-Z**, and **2-U** were confirmed by ^1^H NMR, ^1^H–^1^H COSY, ^13^C NMR spectra, and high-resolution mass spectrometry (HR-MS) (details are given in the Supporting information). The ^1^H NMR spectra of **1-Z** and **1-U** in CDCl_3_ displayed symmetric patterns. The protons at the *K*-regions of **1-Z** give two doublet signals at 8.88 and 8.24 ppm with the other three multiplets in the aromatic region assigned to the protons in the terminal benzene rings. A similar ^1^H NMR spectrum was observed for **1-U** but with separate signals for the diastereotopic TIPS groups. The ^1^H NMR spectra of **2-Z** and **2-U** show a more complex set of signals consistent with the structure (details are given in the Supporting information). In the case of **2-U**, the signals of the inner TIPS group appear to be shifted upfield as a result of anisotropy because this TIPS group sits on top of one of the carbonyls of the quinone.

**Figure 2:**
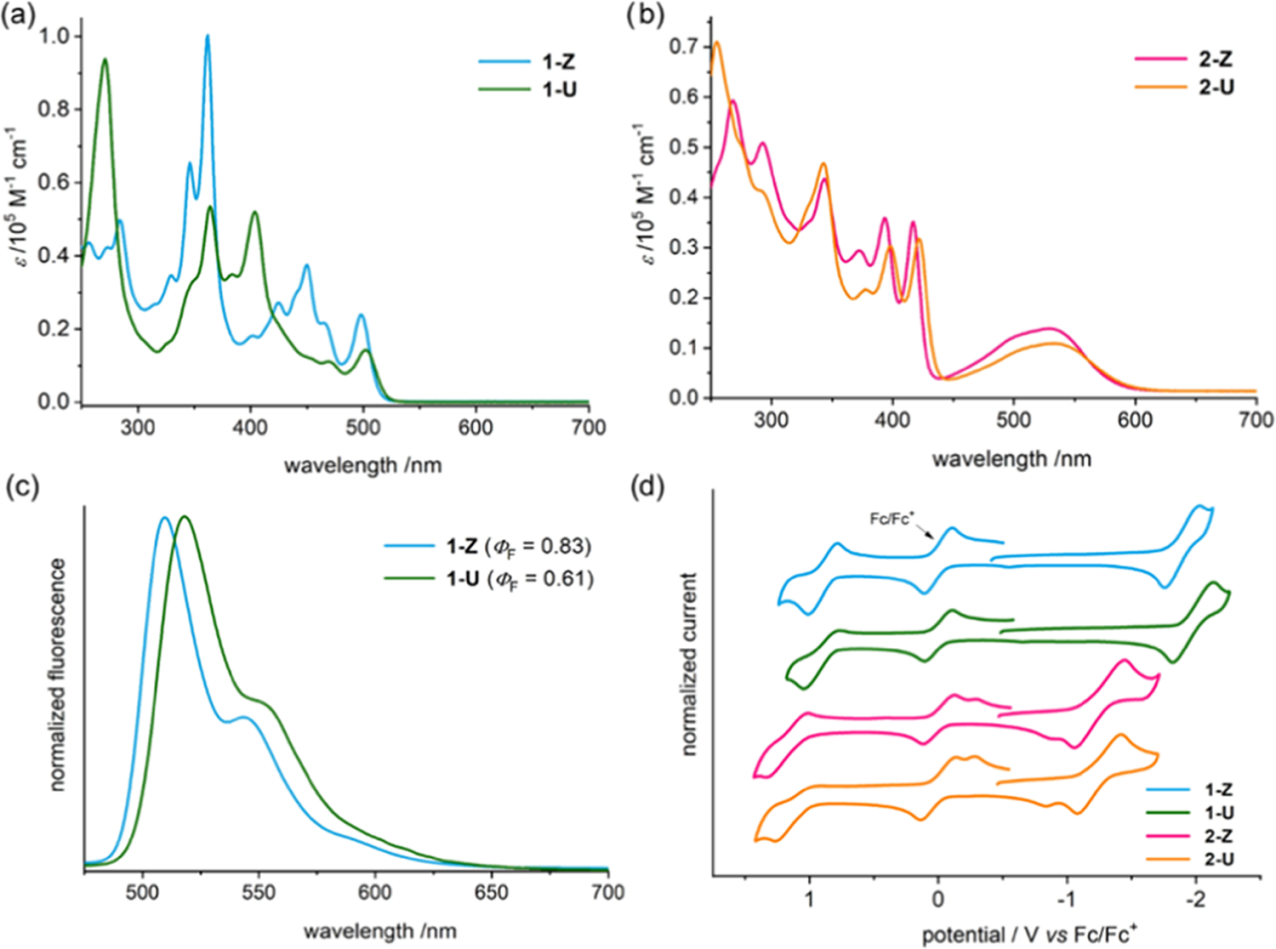
(a) UV/vis absorption spectra of **1-Z** and **1-U** in CH_2_Cl_2_, (b) UV/vis absorption spectra of **2-Z** and **2-U** CH_2_Cl_2_, (c) fluorescence spectra and quantum yields of **1-Z** and **1-U** in CH_2_Cl_2_ (λ_ex_ = 450 nm), and (d) cyclic voltammograms in *o*-dichlorobenzene using *n*Bu_4_NPF_6_ (0.05 M) as an electrolyte (scan rate: 100 mV/s).

The electronic absorption spectra of the naphtophenazines evidenced differences in their optoelectronic properties. In the case of **1-Z** and **1-U**, different absorption spectra were observed, in which the longest-wavelength absorption is slightly red-shifted for **1-U** (Figure 2a). In the case of **2-Z** and **2-U**, a similar pattern was observed that resembles the pattern of the absorption spectrum of **1-U** but with the presence of a broad band between 450 and 600 nm (Figure 2b). This additional band, which is also slightly red-shifted in the case of **2-U**, is consistent with an intramolecular charge transfer process. The optical HOMO–LUMO gaps determined from the onset of the lowest-energy absorption show similar values for the **1-Z** (2.41 eV)/**1-U** (2.38 eV) and **2-Z** (2.12 eV)/**2-U** (2.09 eV) couples (Table 1).

**Table 1:** Summary of Optical, Electrochemical, and Calculated Properties

	λ_abs_^a^ (nm)	λ_em_^a^ (nm)	*E*_g_^opt ^b (eV)	*E*_1/2_^ox^ (V)	*E*_1/2_^red^ (V)	*E*_HOMO_^cv ^c (eV)	*E*_LUMO_^cv ^c (eV)	*E*_g_^cv ^c (V)	*E*_HOMO_^cal. ^d (eV)	*E*_LUMO_^cal. ^d (eV)	*E*_g_^cal. ^d (eV)
**1-Z**	498	510	2.41	0.90	–1.89	–5.44	–3.21	2.23	–5.47	–2.75	2.72
**1-U**	502	518	2.38	0.90	–1.98	–5.44	–3.15	2.29	–5.45	–2.74	2.71
**2-Z**	529		2.12	1.17	–1.25	–5.66	–3.97	1.69	–5.75	–3.39	2.36
**2-U**	532		2.09	1.09	–1.25	–5.58	–3.91	1.67	–5.73	–3.38	2.35

a Absorption and emission were measured in CH_2_Cl_2_.

b Optical band gap was calculated using the equation *E*_g_^opt^ = 1240/λ_offset_, where λ_offset_ is the offset wavelength derived from the lowest-energy absorption band.

c Frontier molecular orbitals and band gaps from cyclic voltammetry were estimated as: *E*_HOMO_^cv^ (eV) = −(*E*_onset_^ox^ – *E*_Fc/Fc^+^_ + 4.8) (eV), *E*_LUMO_^cv^ (eV) = −(*E*_onset_^red^ – *E*_Fc/Fc^+^_ + 4.8) (eV), and *E*_g_^cv^ = *E*_LUMO_^cv^ – *E*_HOMO_^cv^.

d *E*_HOMO_^cal.^ and *E*_LUMO_^cal.^ were calculated at the B3LYP/6-311+G(2d,p)(CH_2_Cl_2_)/B3LYP/6-31G(d,p) level, and *E*_g_^cal.^ was calculated as *E*_g_^cal.^ = *E*_LUMO_^cal.^ – *E*_HOMO_^cal.^.

Sharp and vibronically resolved fluorescence spectra were recorded for **1-Z** (510 nm) and **1-U** (518 nm) with high quantum yields (0.83 and 0.61, respectively) (Figure 2c and Table 1). The high quantum yield for **1-Z** is in agreement with those observed on parent double π-expanded helicenes.^10^ The lower quantum yield observed for **1-U** also agrees with previous reports that describe lower fluorescence quantum yields for twisted systems.^12^**2-Z** and **2-U** showed no fluorescence, which is consistent with a nonemissive intramolecular charge transfer transition (also see the theory section below).

Electrochemical properties were studied by cyclic voltammetry measurements in *o*-dichlorobenzene using *n*Bu_4_NPF_6_ as an electrolyte. The voltammograms in all cases displayed one reduction and two oxidation processes (Figure 2d). The redox potentials are summarized in Table 1. Compounds **1-Z** and **1-U** showed an identical oxidation potential at +0.9 V and a reduction potential at around −1.9 V, which is slightly more negative for **1-U**. Whereas, **2-Z** and **2-U** displayed a very similar oxidation potential at around +1.1 V and the same reduction potential at −1.25 V. The electrochemical highest-occupied molecular orbital (HOMO) (ionization potentials) and the lowest-unoccupied molecular orbital (LUMO) (electron affinities) were estimated from the onset of the first oxidation and reduction waves, respectively (Table 1). The HOMO levels are the same for **1-Z** and **1-U** (−5.44 eV), whereas the HOMO level for **2-Z** (−5.66 eV) is slightly lower than that of **2-U** (−5.58 eV). The LUMO levels are very similar in the case of **1-Z** (−3.21 eV) and **1-U** (−3.15 eV). The energy of LUMO drops when a quinone is present in the aromatic framework **2-Z** (−3.97 eV) and **2-U** (−3.91 eV). The electrochemical HOMO–LUMO gaps of **1-Z** (2.23 eV)/**1-U** (2.29 eV) and **2-Z** (1.69 eV)/**2-U** (1.67 eV) couples show the same trends as the optical HOMO–LUMO gaps.

**Figure 3:**
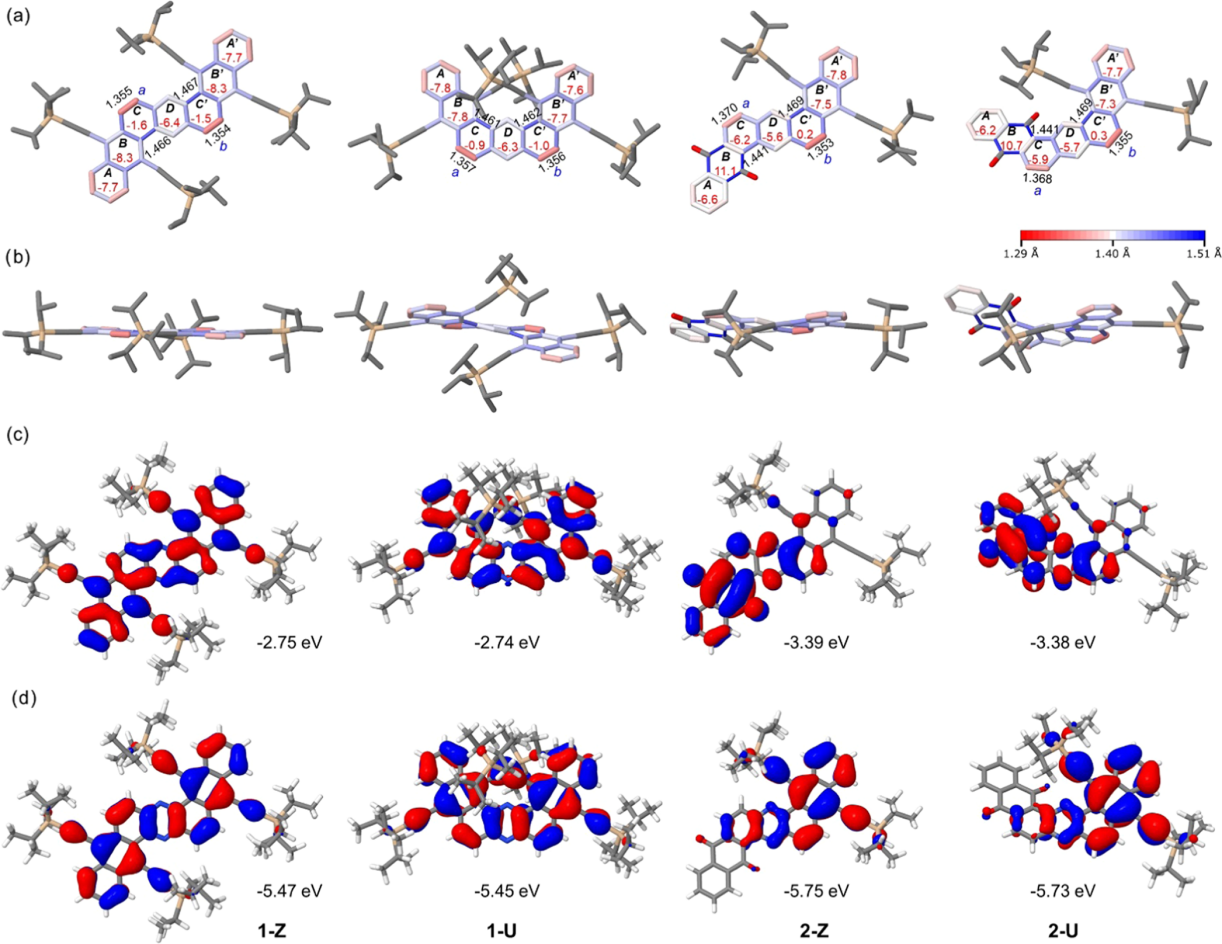
(a) Top view and (b) side view of the optimized geometries with selected bond length (Å) and nuclear independent chemical shift (NICS) (0) values (in red) calculated at the B3LYP/6-31G(d,p) level. Bonds are rendered in a color continuum ranging from red (1.29 Å) to white (1.40 Å) to blue (1.51 Å) so that Clar’s aromatic sextets are lighter/whiter colors and localized double and single bonds are red and blue, respectively. (c) LUMO and (d) HOMO orbitals calculated at the B3LYP/6-31G(d,p) level.

After several attempts, we were unable to grow single crystals suitable for X-ray diffraction, so we relied on calculations to get an insight into their structures. The optimized geometries were calculated at the B3LYP/6-31G(d,p) level (Figures 3a and S1). For comparison, optimized structures with the same Hamiltonian augmented by a dispersion correction were also computed yielding almost identical results (Figure S2 and Table S1). Dinaphthophenazine **1-Z** is virtually planar with small twists, in agreement with a previously reported crystal structure.^8^ Conversely, **1-U** adopts a helical structure due to the steric hindrance resulting from the bulky TIPS groups in the inner rim. The structure of **1-U** shows large torsion angles along the BCD/B′C′D′ rings (21.5 and 20.7°, respectively, Figure S1) that together produce a helix angle of 42.2°. This helical structure and the helix angle value is consistent with the structures and helix angles observed on a parent double π-expanded helicene (40.6°)^10^ and with those of a similar family of less-strained π-expanded helicenoids (28°).^13^ In the case of the quinone series, dinaphthophenazine **2-Z** is not as planar as **1-Z**. Similarly, compound **2-U** also shows a helical structure with torsion angles along the BCD/B′C′D′ rings (15.0 and 16.2°, respectively) that are lower than those observed in **1-U**. This is because of the lower strained structure as a result of only one TIPS–acetylene group in the inner rim, which generates a smaller helix angle of 31.2°. The conformational stability of the **1-U** and **2-U** was investigated at the xtb-GFN1 level with a metadynamics procedure. In the case of **1-U**, no evidence of racemization was observed, as neither metadynamics nor nudge elastic band methodologies produced a chemically sensible path for this process. This is consistent with the large overlap between the two TIPS groups that practically locks the structure. Whereas, in the case of **2-U**, the racemization is possible, as illustrated by a relatively low barrier (96 kJ/mol, Figure S3).

Based on the bond length analysis and on the local aromaticity indicated by the nuclear independent chemical shift (NICS) values (Figure 3a), the dominant resonance structures are best represented by Clar’s sextet rule. In the case of **1-Z** and **1-U**, the dominant electronic structure consists of two *K*-regions (C and C′) with a double bond character (bond lengths ∼1.35 Å), two naphthalene groups (AB and A′B′), and a pyrazine (D) group. For instance, the lowest NICS (0) values for **1-Z** and **1-U** (indicated in each ring of Figure 3a) were found on the naphthalene and pyrazine groups, while NICS (0) values close to zero were found on the rings of the *K*-regions. Meanwhile, in the case of **2-Z** and **2-U**, the dominant electronic structure consists of two *K*-regions (C and C′) with a double bond character (bond lengths ∼1.35–1.37 Å), a naphthalene group (A′B′), a benzene group (A), a pyrazine (D) group, and a quinone (B) group. In the case of **2-Z** and **2-U**, the lowest NICS (0) values (indicated in each ring of Figure 3a) were found on the naphthalene (A′B′), pyrazine (D), and benzene (A) groups. One of the *K*-region rings shows again a similar NICS (0) value approaching zero, whereas the *K*-region ring next to the quinone ring shows a more negative value. The quinone rings (B′) display positive NICS (0) values, indicating their antiaromatic character.

Theoretical calculations (B3LYP/6-311+G(2d,p)(CH_2_Cl_2_)/B3LYP/6-31G(d,p)) were carried out to shine additional light on the optoelectronic and redox properties of the dinaphthophenazines. The calculated energy gaps show the same trends observed in the optical and electrochemical gaps (Table 1). According to time-dependent density functional theory (TD-DFT) calculations, the longest-wavelength absorptions of all of these compounds were mainly attributed to the HOMO → LUMO transitions (Table S2). The HOMO and LUMO orbitals for **1-Z** and **1-U** are thoroughly delocalized along the dinaphtophenazine core (Figure 3b,c), whereas the HOMOs of **2-Z** and **2-U** are mainly located on the triisopropylsilylethynyl-substituted naphtophenazine side. The electronic distribution of the HOMO and the LUMO on different sides of the dinaphthophenazine core in the case of **2-Z** and **2-U** implies intramolecular charge transfer for this excitation.

## Conclusions

In summary, we have described the synthesis of planar and helical dinaphthophenazines by cyclocondensation reactions. This was achieved by condensation of anthracene-1,2-dione **4**, with different diamines. Due to the steric interactions raised by the bulky TIPS groups, both U-shaped dinaphthophenazines **1-U** and **2-U** show helicenoid structures, whereas the core of the Z-shaped dinaphthophenazines **1-Z** and **2-U** remains virtually planar. Optoelectronic characterization reveals different absorption patterns for **1-Z** and **1-U** but similar fluorescence properties with high quantum yields (0.83 and 0.61, respectively). A different behavior was observed on the quinone containing **2-Z** and **2-U**, which shows similar absorption patterns and the presence of an additional band consistent with a nonemissive intramolecular charge transfer process. Theoretical calculations are consistent with experimental observations and indicate that the presence of the fused quinone on one of the sides strongly polarizes the dinaphthophenazine core favoring the charge transfer process. Overall, this work provides a new route for the synthesis of dinaphthophenazines and also of new valuable precursors that can be used in the synthesis of other and more complex PAHs and nanographenes.

## Experimental Section

### General Information

Commercially available solvents and reagents were used without further purification unless otherwise noted. Column chromatography was carried out using a Silica gel 60 from Scharlab. UV/visible absorption spectra were recorded on a Perkin-Elmer Lambda 950 spectrometer. Fluorescence spectra were registered on a LS55 Perkin-Elmer Fluorescence spectrometer.^1^H and ^13^C NMR spectra were recorded on a Bruker Avance 400 and 500 spectrometers at 298 K using partially deuterated solvents as internal standards. High-resolution atmospheric-pressure-chemical-ionization time-of-flight mass-spectrometry (HR-APCI-TOF-MS) measurements were carried out in the General Services of the University of the Basque Country (SGIker) in a Thermo LCQ Advantage using positive-ion mode by Dr. Alicia Sánchez. High-resolution matrix-assisted laser desorption ionization mass spectrometry (HR-MALDI-TOF-MS) measurements were carried out in CIC Biomagune in an Ultraflex III (Bruker Daltonics) MALDI-TOF (frequency-tripled (355 nm) Nd:YAG laser) by Dr. Javier Calvo. Cyclic voltammetry measurements were carried out on a Princeton Applied Research Parstat 2273 in a three-electrode single compartment cell with a glassy carbon disc working electrode, a platinum wire counter electrode, and a silver wire pseudoreference electrode. All of the potential values are reported as *E*_1/2_ = (*E*_p_^a^ + *E*_p_^c^)/2 in V versus the redox potential of the ferrocene/ferrocenium couple. 6,11-Bis(2-(triisopropylsilyl)ethynyl)anthra[2,1-*c*][1,2,5]thiadiazole (**5**)^10^ and 2-hydroxy-9,10-bis(triisopropylsilylethynyl)anthracene (**3**)^14^ were prepared according to reported procedures, respectively.

#### Synthesis of 9,10-Bis(2-(triisopropylsilyl)ethynyl)anthracene-1,2-dione (**4**)

A solution of **3** (0.215 g, 0.388 mmol) in dry tetrahydrofuran (THF) (20 mL) was added to a suspension of phenylseleninic anhydride (0.21 g, 70%, 0.582 mmol) in dry THF (50 mL) at 50 °C in an oil bath under N_2_. The reaction was stirred at room temperature for 24 h. Then, the mixture was diluted with dichloromethane and washed with aqueous NaHCO_3_. The organic layer was washed with water and brine and dried over anhydrous Na_2_SO_4_. After removal of the solvents, the crude product was purified with column chromatography on silica to obtain **4** (0.21 g, 94%) as red solids. ^1^H NMR (400 MHz, CDCl_3_, ppm) δ 8.75–8.73 (m, 1H), 8.48–8.46 (m, 1H), 8.39 (d, *J* = 10.2 Hz, 1H), 7.78–7.70 (m, 2H), 6.63 (d, *J* = 10.2 Hz, 1H), 1.27–1.21 (m, 42H).^13^C{1H} NMR (101 MHz, CDCl3, ppm) δ 181.1, 177.9, 144.3, 135.0, 134.2, 132.6, 131.1, 130.1, 129.9, 129.4, 128.4, 128.2, 126.6, 123.4, 111.6, 107.6, 102.6, 100.6, 18.9, 11.6, 11.5. HR-APCI-TOF-MS: *m*/*z* calcd for C_36_H_48_O_2_Si_2_ [M + H]^+^, 569.3271, found 569.3268.

#### Synthesis of **1-Z** and **1-U**

LiAlH_4_ (5.0 mL, 4 M in diethyl ether, 20.0 mmol) was added dropwise under N_2_ to a round-bottom flask charged with a solution of **5** (238.5 mg, 0.4 mmol) in dry diethyl ether (50 mL) at 0 °C. Then, the reaction mixture was stirred at room temperature overnight. Then, the reaction was quenched with saturated NH_4_Cl (aq) and extracted with dichloromethane. The combined organic layers were washed with water and brine and dried over anhydrous Na_2_SO_4_. After the removal of the solvents, the residue was used directly in the next step. A mixture of acetic acid/chloroform (30:30 mL) was added to a Schlenk tube charged with the residue of the previous step and with compound **4** (113.7 mg, 0.2 mmol). The reaction was stirred at room temperature for 48 h and then heated to reflux in an oil bath for 48 h. After cooling to room temperature, the reaction was quenched with saturated NH_4_Cl (aq), and the mixture was extracted with dichloromethane. The combined organic layers were washed with brine and dried over Na_2_SO_4_. After removal of the solvents, the crude product was purified with column chromatography on a silica gel with hexane/dichloromethane (DCM) as an eluent to obtain **1-Z** (70.6 mg, 32%) and **1-U** (80.0 mg, 36%).

**1-Z**: ^1^H NMR (500 MHz, CDCl_3_, ppm) δ 9.23–9.09 (m, 2H), 8.88 (d, *J* = 9.5 Hz, 2H), 8.78–8.71 (m, 2H), 8.23 (d, *J* = 9.5 Hz, 2H), 7.85–7.70 (m, 4H), 1.32–1.30 (m, 84H).^13^C{1H} NMR (125 MHz, CDCl_3_, ppm) δ 142.6, 141.3, 134.52, 133.49, 133.2, 130.9, 129.2, 128.7, 128.4, 128.1, 127.8, 127.4, 119.8, 119.7, 107.8, 106.2, 105.6, 103.3, 19.2, 19.0, 12.0, 11.7. HR-MALDI-TOF-MS: *m*/*z* calcd for C_72_H_96_N_2_Si_4_ [M + H]^+^, 1101.6727, found 1101.6808.

**1-U**: ^1^H NMR (500 MHz, CDCl_3_, ppm) δ 8.95–8.89 (m, 2H), 8.78–8.70 (m, 4H), 7.94 (d, *J* = 9.3 Hz, 2H), 7.77–7.71 (m, 4H), 1.35–1.28 (m, 42H), 0.74–0.69 (m, 18H), 0.63–0.60 (m, 24H).^13^C{1H} NMR (125 MHz, CDCl_3_, ppm) δ 142.4, 138.7, 134.0, 133.2, 131.5, 129.9, 128.5, 127.9, 127.8, 127.5, 127.2, 120.9, 118.7, 105.7, 105.0, 103.8, 103.1, 19.1, 18.51, 18.47, 11.7, 11.5. HR-MALDI-TOF-MS: *m*/*z* calcd for C_72_H_96_N_2_Si_4_ [M + H]^+^, 1101.6727, found 1101.6810.

#### Synthesis of **2-Z** and **2-U**

A Schlenk tube was charged with **4** (0.114 g, 0.2 mmol) and 1,2-diaminoanthraquinone **7** (57.13 mg, 0.24 mmol) under N_2_. Then, a mixture of acetic acid and chloroform (15:15 mL) was added. The mixture was heated to reflux in an oil bath and stirred for 2 days. After cooling to room temperature, the mixture was diluted with dichloromethane, washed with water and brine, and dried over Na_2_SO_4_. After evaporation of the solvents, purification of the residue with column chromatography afforded **2-Z** (47.5 mg, 31%) and **2-U** (67.2 mg, 44%) as red solids.

**2-Z**: ^1^H NMR (500 MHz, CDCl_3_, ppm) δ 9.11–9.09 (m, 1H), 8.85 (d, *J* = 9.6 Hz, 1H), 8.75–8.72 (m, 2H), 8.43–8.41 (m, 1H), 8.33–8.32 (m, 1H), 8.11 (d, *J* = 9.6 Hz, 1H), 7.89–7.78 (m, 5H), 1.30–1.27 (m, 42H). ^13^C{1H} NMR (125 MHz, CDCl_3_, ppm) δ 183.8, 146.9, 145.0, 142.8, 139.9, 135.9, 135.6, 135.2, 134.7, 134.5, 134.2, 133.8, 133.7, 133.2, 132.2, 129.1, 128.9, 128.6, 128.5, 128.3, 127.6, 126.7, 125.9, 121.3, 120.7, 108.8, 106.4, 105.8, 102.4, 19.1, 19.0, 11.9, 11.6. HR-MALDI-TOF-MS: *m*/*z* calcd for C_50_H_52_N_2_O_2_Si_2_ [M + H]^+^, 771.3801, found 771.3751.

**2-U**: ^1^H NMR (500 MHz, CDCl_3_, ppm) δ 9.04–9.01 (m, 1H), 8.78–8.69 (m, 3H), 8.53 (d, *J* = 8.8 Hz, 1H), 8.37–8.34 (m, 2H), 7.88–7.76 (m, 5H), 1.32–1.25 (m, 21H), 0.98–0.92 (m, 21H).^13^C{1H} NMR (125 MHz, CDCl_3_, ppm) δ 183.8, 181.8, 145.9, 144.8, 144.1, 137.5, 135.1, 135.0, 134.8, 134.7, 134.4, 133.8, 133.6, 133.2, 132.2, 129.8, 129.3, 129.0, 128.8, 128.3, 127.7, 127.5, 127.5, 126.7, 121.8, 120.1, 105.9, 105.3, 104.7, 102.4, 19.0, 18.8, 11.7, 11.6. HR-MALDI-TOF-MS: *m*/*z* calcd for C_50_H_52_N_2_O_2_Si_2_ [M + H]^+^, 771.3801, found 771.3724.

## Supporting Information Available

Optimized geometries, NMR spectra, and calculations (PDF)
