# Supplementary material for: Planar and Helical Dinaphthophenazines
Source: J Org Chem. 2022 May 26;87(12):7635–42. doi: 10.1021/acs.joc.2c00129 (PMC9207929; doi:10.1021/acs.joc.2c00129)
Supplement: Supplementary file 1 — jo2c00129_si_001.pdf [file jo2c00129_si_001.pdf]

Supporting Information
for

Planar and Helical Dinaphthophenazines

Fengkun Chen,[a] Manuel Melle-Franco,[b] and Aurelio Mateo-Alonso*[a][c]

[a] POLYMAT, University of the Basque Country UPV/EHU. Avenida de Tolosa 72, 20018 Donostia-San Sebastian (Spain)

[b] CICECO—Aveiro Institute of Materials. Department of Chemistry, University of Aveiro. 3810-193 Aveiro (Portugal)

[c] Ikerbasque, Basque Foundation for Science, 48009 Bilbao (Spain)

Contents

1. Calculations S1

2. NMR spectra S6

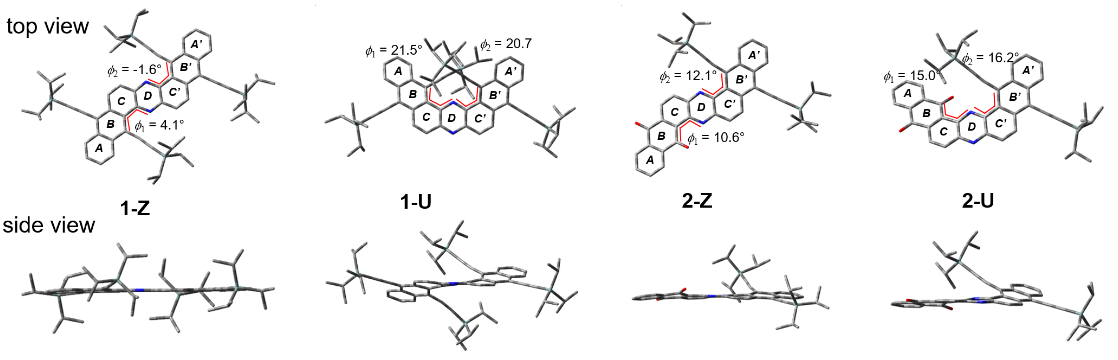

Figure S1. Optimized geometries (top: top view; bottom: side view) with selected torsion angles calculated at the B3LYP/6-31G(d,p) level (hydrogen atoms are omitted for clarity).

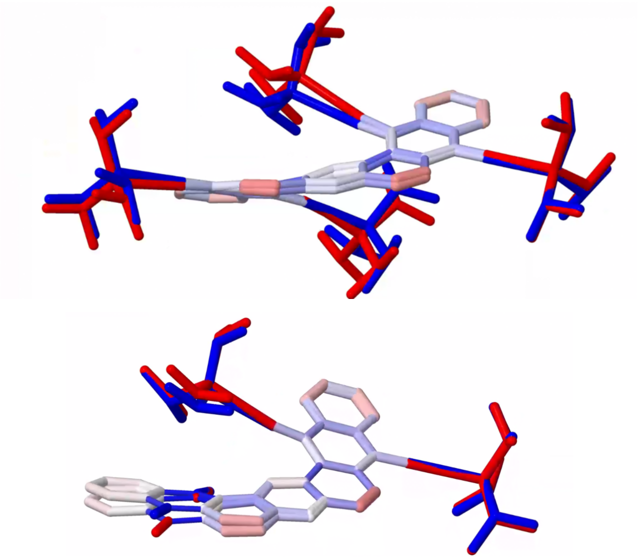

Figure S2. Comparison of minimized U dimer structures at the B3LYP/6-31G(d,p) level with dispersion correction (D3BJ) in blue and without dispersion correction in red. The RSMD displacements are 0.64 A and 0.34 A respectively. Csp2-Csp2 bonds are rendered in a colour continuum ranging from red (1.29 A) to white (1.40 A) to blue (1.51 A)

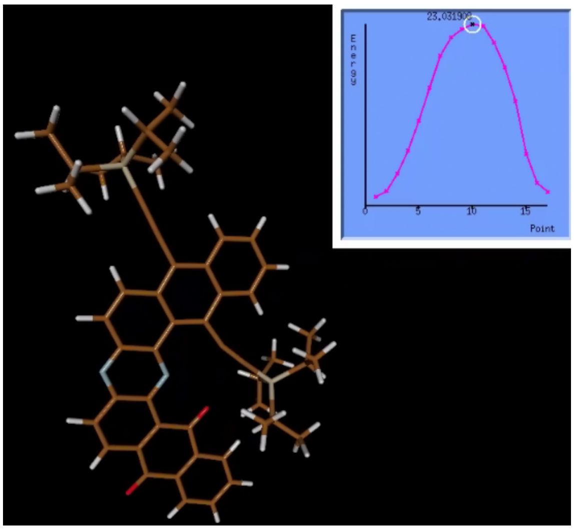

Figure S3. The energy of racemization of **2-U** (kcal/mol).

Table S1. Orbitals eigenvalues at different levels. All energies are eV.

	LUMO	HOMO	Gap
**1-U**[a]	-2.44	-5.18	2.75
**1-U**[b]	-2.47	-5.22	2.76
**2-U**[a]	-3.10	-5.49	2.39
**2-U**[b]	-3.13	-5.50	2.37

[a] Calculated at the B3LYP/6-31G(d,p) level; [b]Calculated at the B3LYP/6-31G(d,p)-D3BJ level.

Table S2. First transitions computed at B3LYP/6-311+G(2d, p) (CH_2Cl_2) /B3LYP/6-31G(d,p) level.

System	Excitation energy [eV]	Wavelength [nm]	*f*	Contributions
**1-Z**	2.37	524	0.31	HOMO→LUMO (94%)
**1-U**	2.35	527	0.07	HOMO→LUMO (98%)
**2-Z**	1.99	624	0.25	HOMO→LUMO (98%)
**2-U**	1.93	642	0.16	HOMO→LUMO (99%)

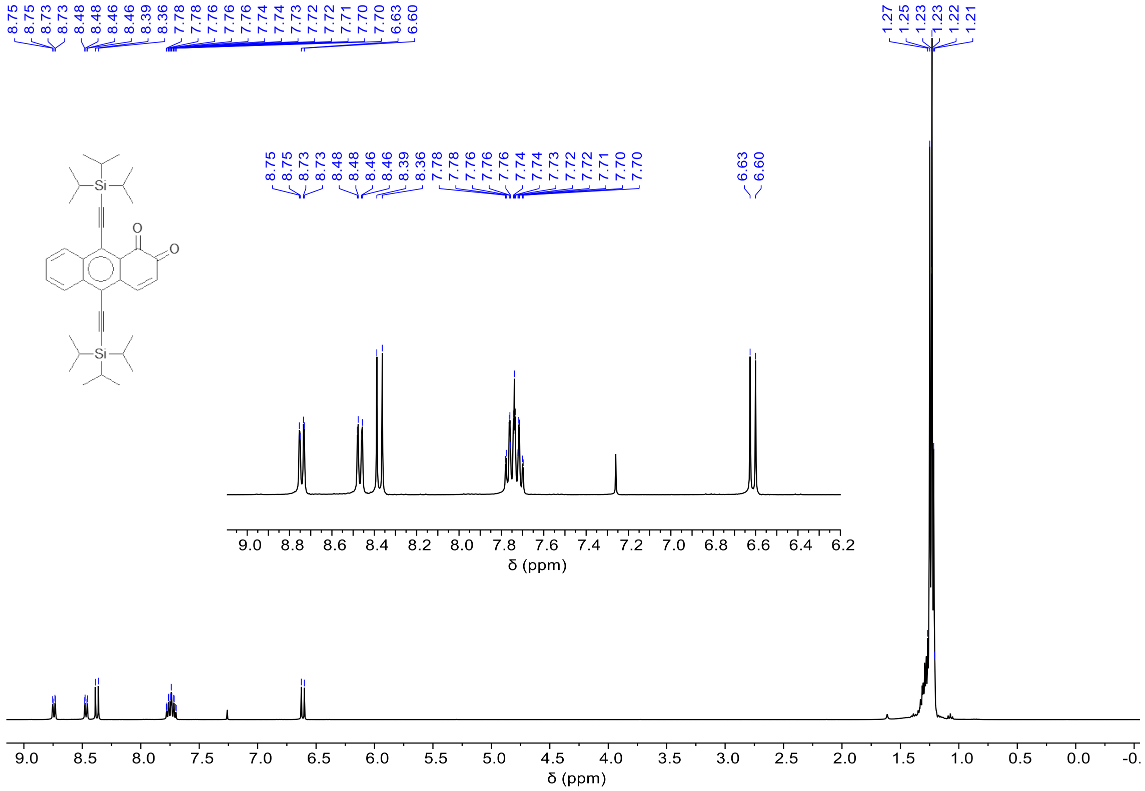

Figure S4. ^{1}H NMR spectrum of **4** in $CDCl_3$ at room temperature (400 MHz).

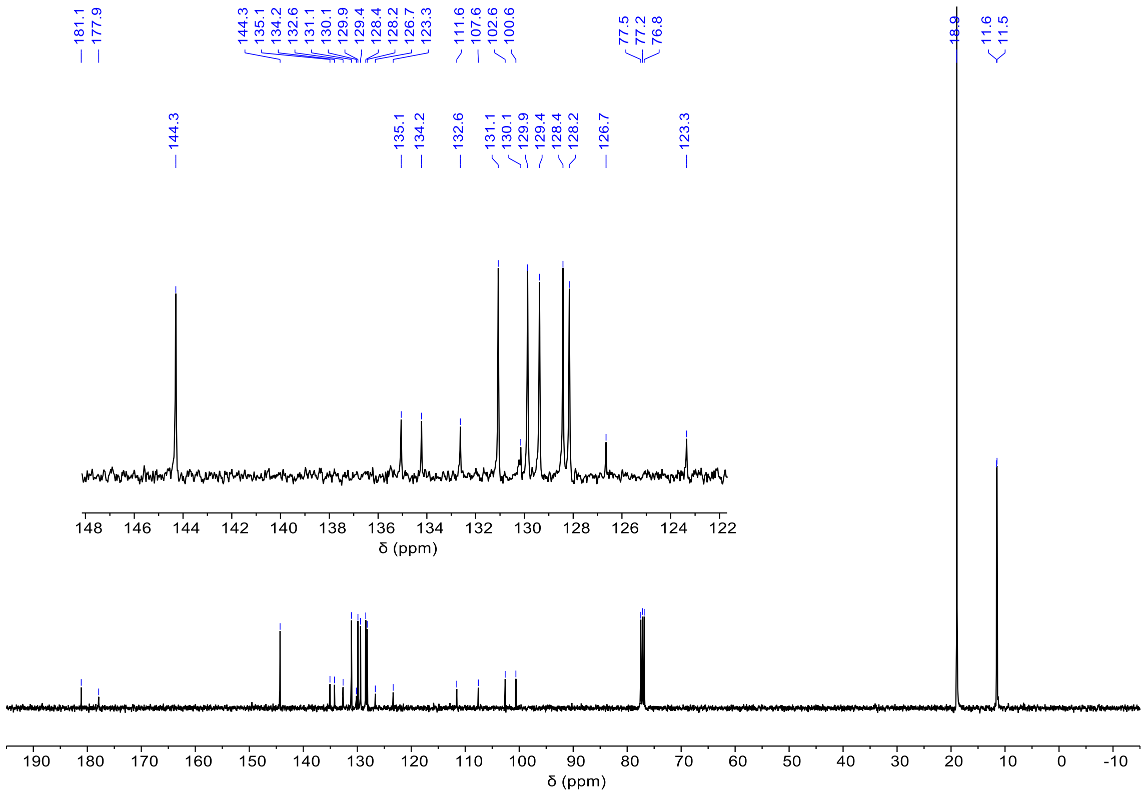

Figure S5. ^{13}C NMR spectrum of **4** in $CDCl_3$ at room temperature (101 MHz).

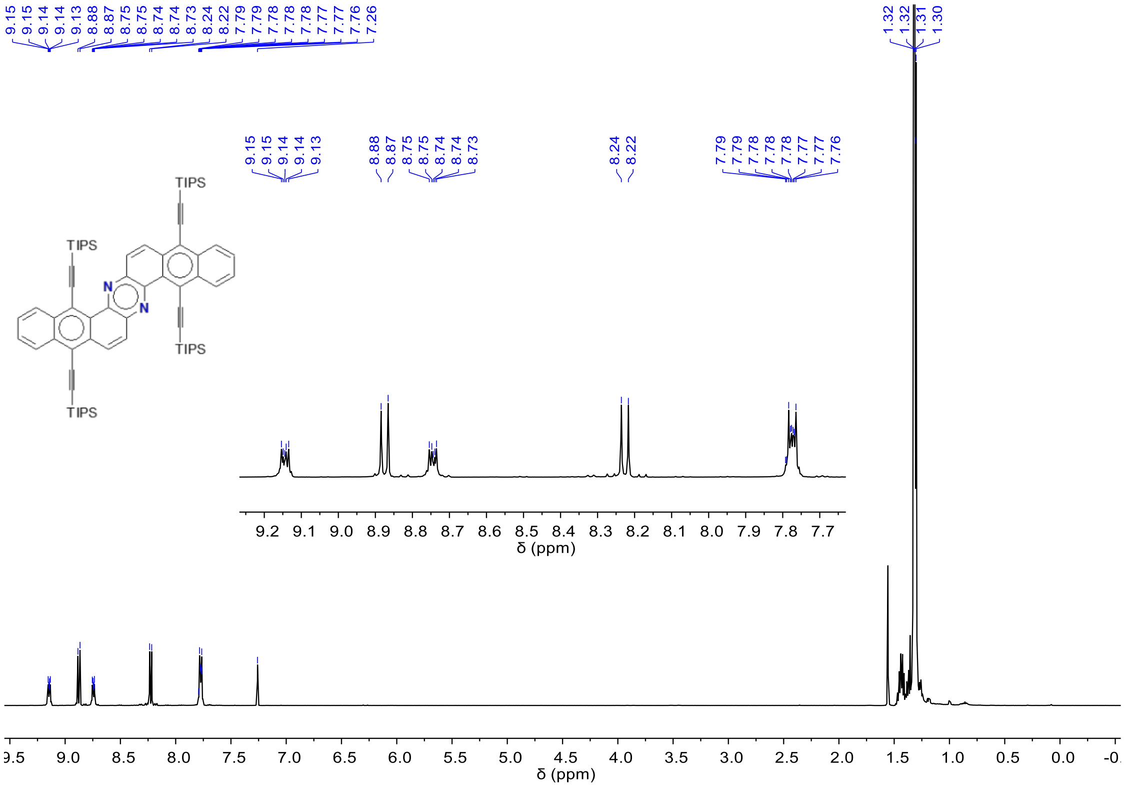

Figure S6. ^1H NMR spectrum of **1-Z** in $CDCl_3$ at room temperature (500 MHz).

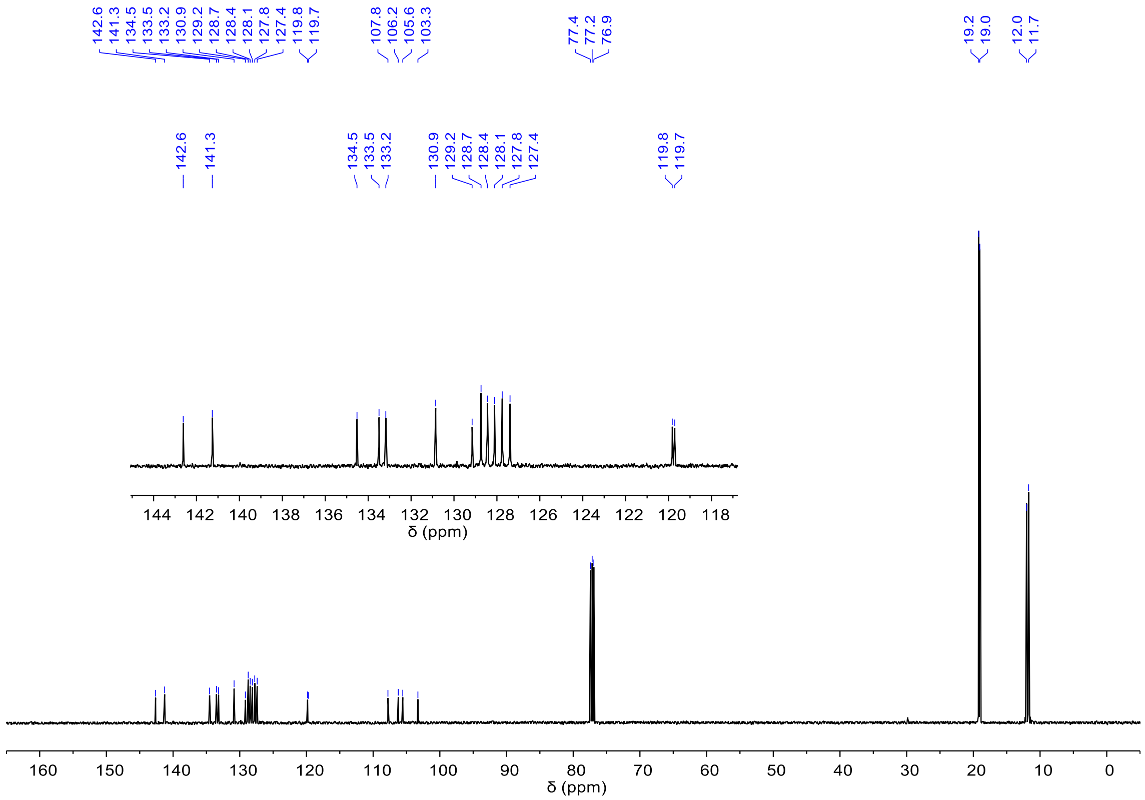

Figure S7. ^{13}C-NMR spectrum of **1-Z** in $CDCl_3$ at room temperature (125 MHz).

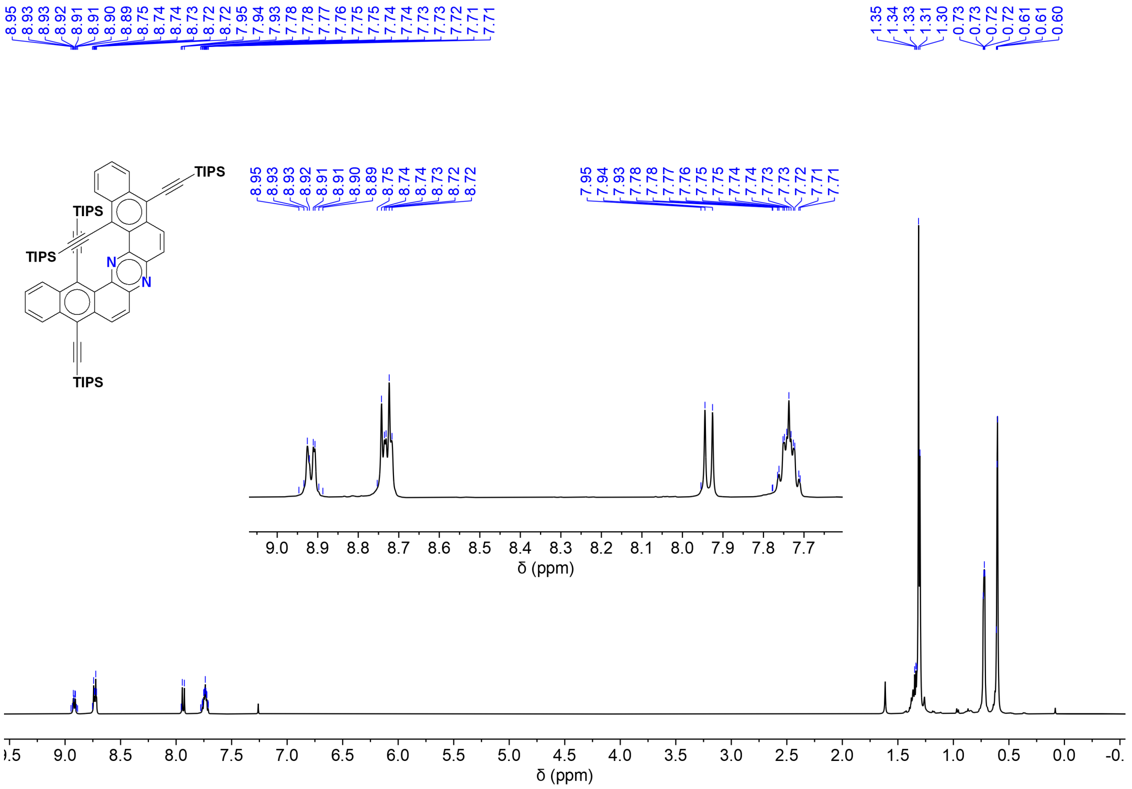

Figure S8. ^{1}H NMR spectrum of **1-U** in $CDCl_3$ at room temperature (500 MHz).

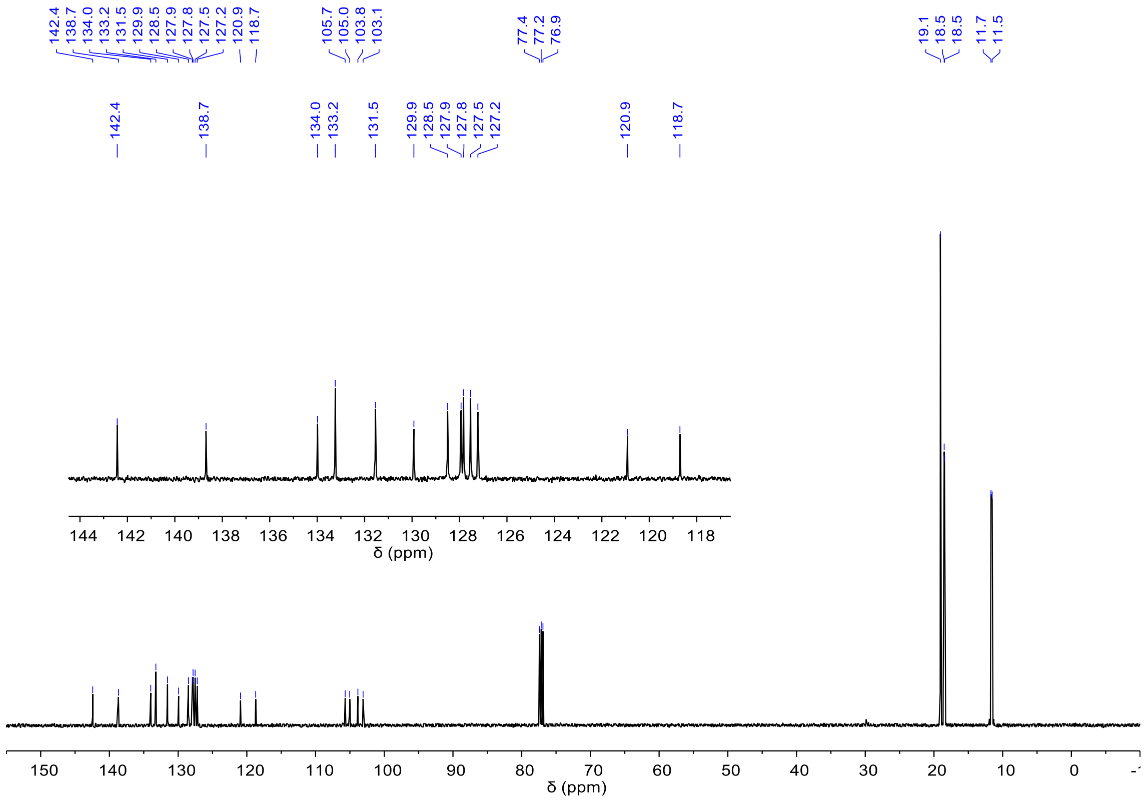

Figure S9. ^{13}C-NMR spectrum of **1-U** in $CDCl_3$ at room temperature (125 MHz).

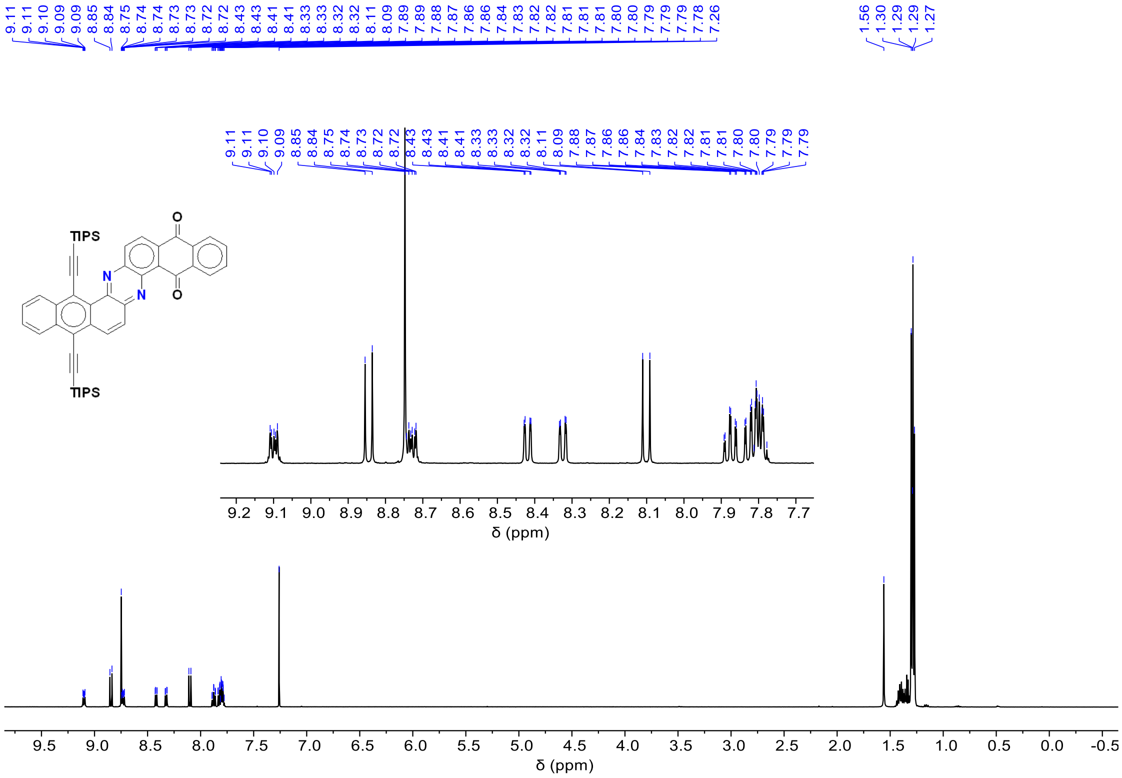

Figure S10. ^{1}H NMR spectrum of **2-Z** in $CDCl_3$ at room temperature (500 MHz).

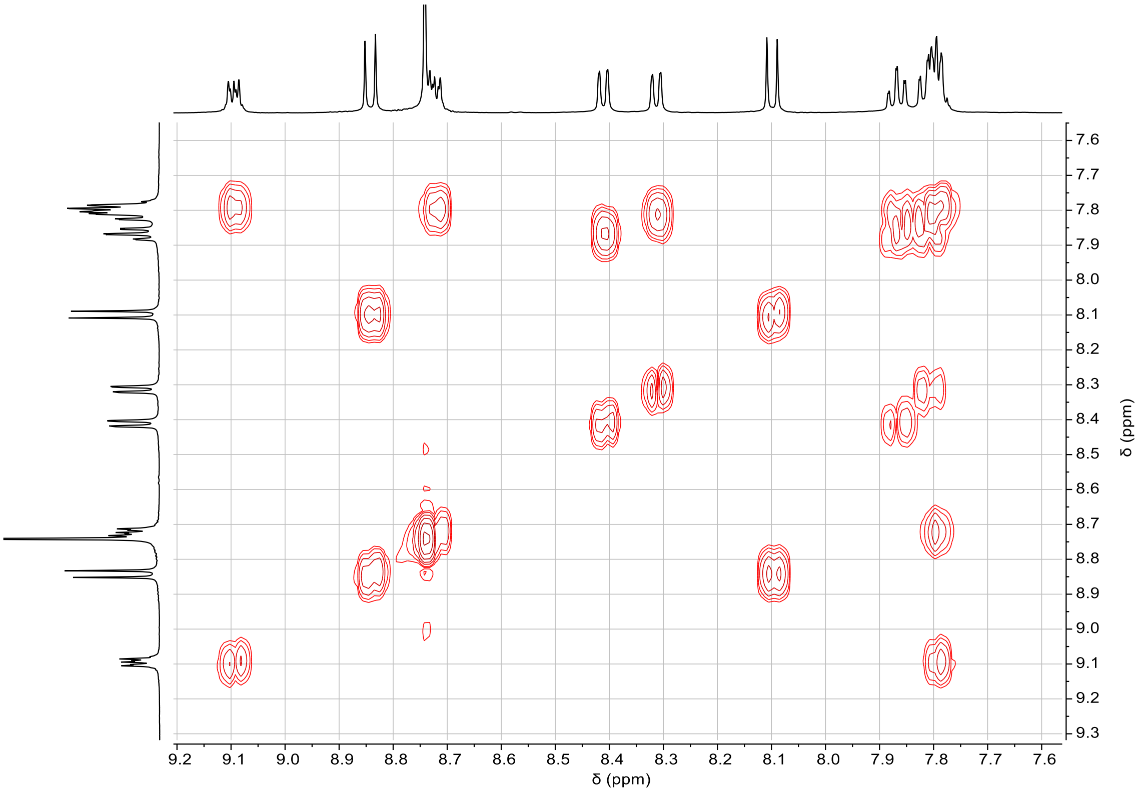

Figure S11. ^{1}H-^{1}H COSY chart of **2-Z** in $CDCl_3$ at room temperature (500 MHz).

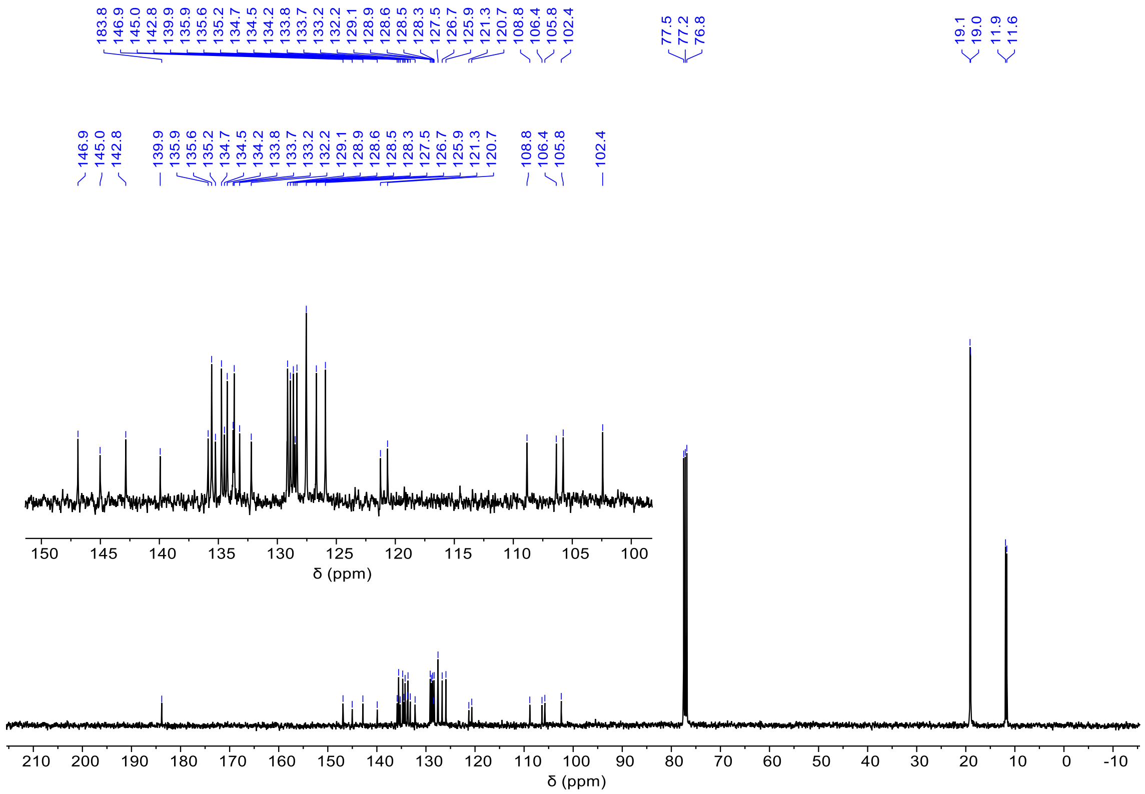

Figure S12. ^{13}C NMR spectrum of **2-Z** in $CDCl_3$ at room temperature (125 MHz).

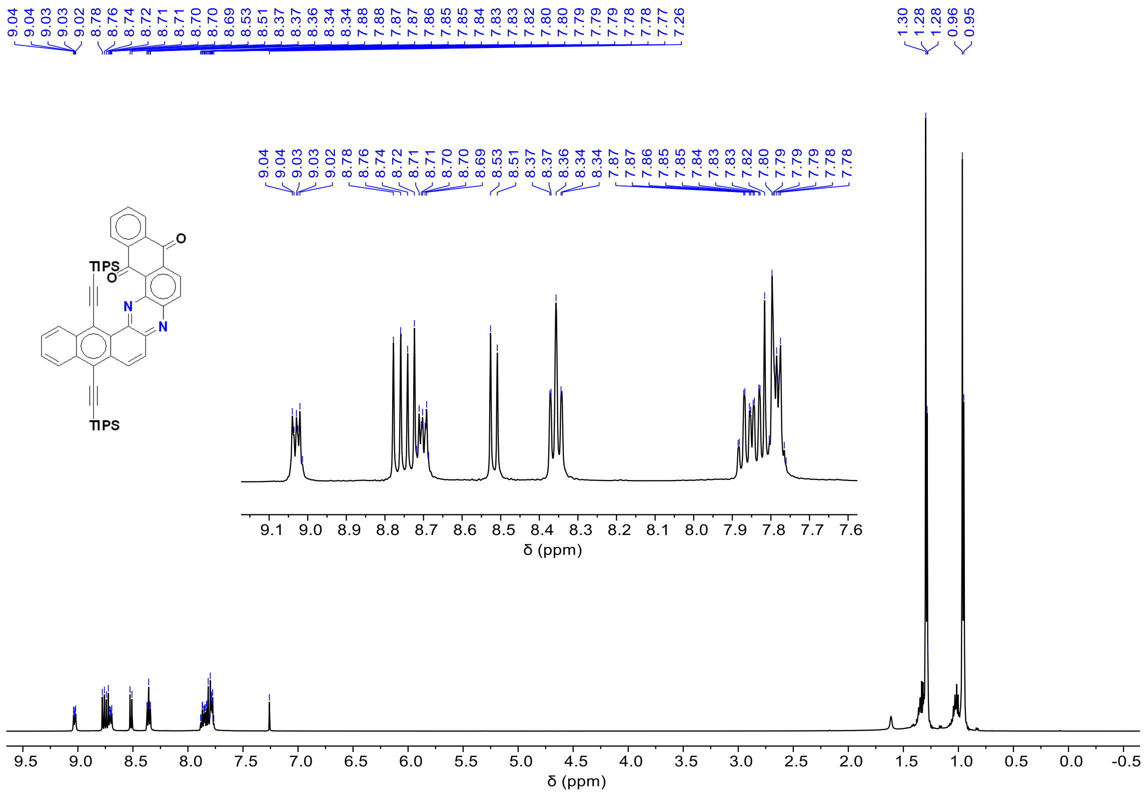

Figure S13. ^{1}H NMR spectrum of **2-U** in $CDCl_3$ at room temperature (500 MHz).

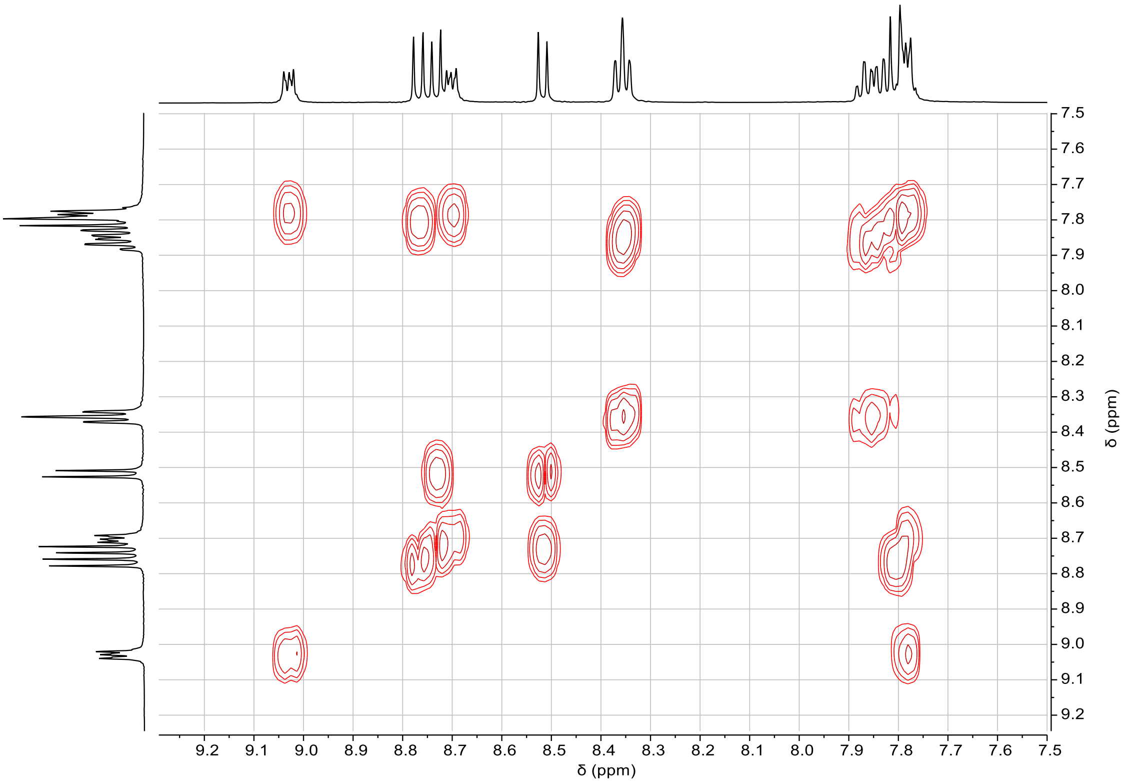

Figure S14. ^1H-^1H COSY chart of **2-U** in $CDCl_3$ at room temperature (500 MHz).

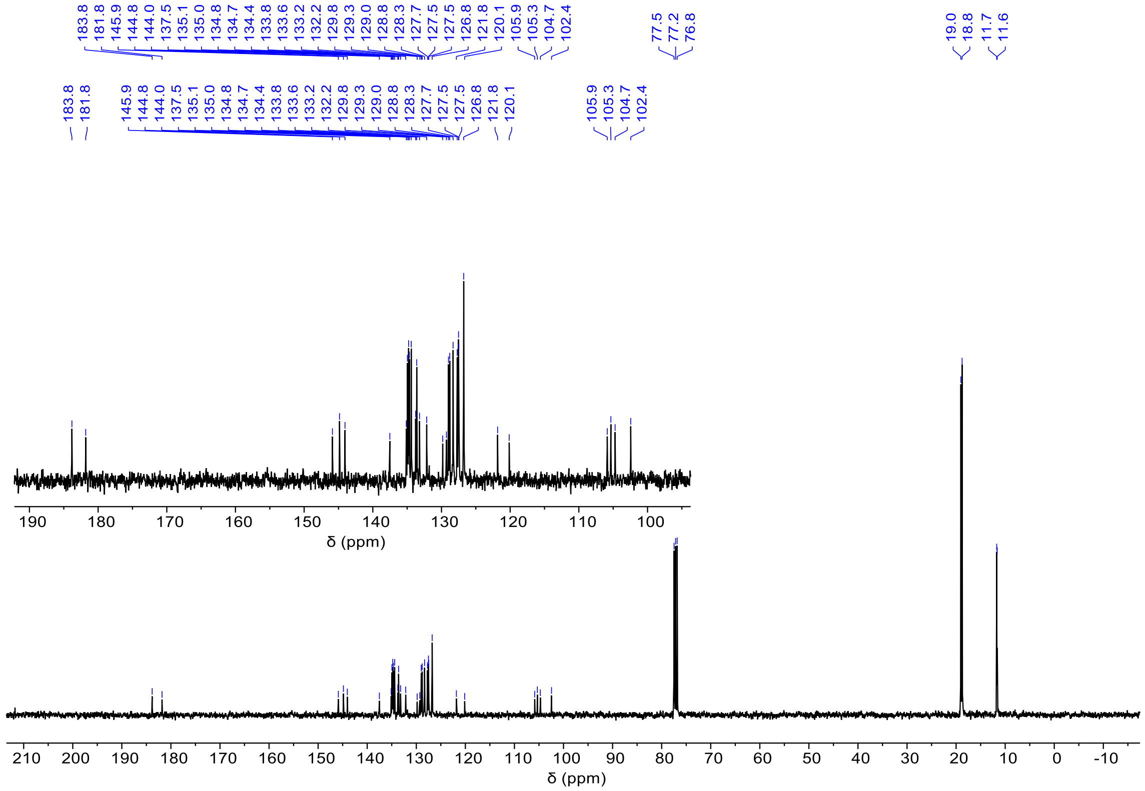

Figure S15. ^{13}C NMR spectrum of **2-U** in $CDCl_3$ at room temperature (125 MHz).
